# Superoxide Dismutase Premodulates Oxidative Stress in Plastids for Protection of Tobacco Plants from Cold Damage Ultrastructure Damage

**DOI:** 10.3390/ijms25105544

**Published:** 2024-05-19

**Authors:** Ekaterina N. Baranova, Neonila V. Kononenko, Pyotr V. Lapshin, Tatiana L. Nechaeva, Marat R. Khaliluev, Natalia V. Zagoskina, Elena A. Smirnova, Natalya O. Yuorieva, Galina N. Raldugina, Inna A. Chaban, Ludmila V. Kurenina, Alexander A. Gulevich

**Affiliations:** 1 All-Russia Research Institute of Agricultural Biotechnology, Timiryazevskaya St. 42, 127550 Moscow, Russiamarat131084@rambler.ru (M.R.K.); kinggobi@yandex.ru (E.A.S.); inna_chaban@rambler.ru (I.A.C.); ludmila.kur2208@gmail.com (L.V.K.); 2 N.V. Tsitsin Main Botanical Garden of Russian Academy of Sciences, 127276 Moscow, Russia; 3 Moscow K.A. Timiryazev Agricultural Academy (RSAU-MTAA), Russian State Agrarian University, Timiryazevskaya 49, 127434 Moscow, Russia; 4 Timiryazev Institute of Plant Physiology, Russian Academy of Sciences, Botanicheskaya St. 35, 127276 Moscow, Russianechaevatatyana.07@yandex.ru (T.L.N.); nzagoskina@mail.ru (N.V.Z.);; 5 Biology Faculty, Lomonosov Moscow State University, Leninskie Gory 1, Building 12, 119991 Moscow, Russia; 6 Department of Biology, MSU-BIT University, Shenzhen 518172, China

**Keywords:** superoxide dismutase, ultrastructure, plastid grana lamellae, chloroplast structural damage, *Nicotiana tabacum* L., ROS

## Abstract

ROS-dependent induction of oxidative damage can be used as a trigger initiating genetically determined non-specific protection in plant cells and tissues. Plants are potentially able to withstand various specific (toxic, osmotic) factors of abiotic effects, but do not have sufficient or specific sensitivity to form an adequate effective response. In this work, we demonstrate one of the possible approaches for successful cold acclimation through the formation of effective protection of photosynthetic structures due to the insertion of the heterologous *FeSOD* gene into the tobacco genome under the control of the constitutive promoter and equipped with a signal sequence targeting the protein to plastid. The increased enzymatic activity of superoxide dismutase in the plastid compartment of transgenic tobacco plants enables them to tolerate the oxidative factor of environmental stresses scavenging ROS. On the other hand, the cost of such resistance is quite high and, when grown under normal conditions, disturbs the arrangement of the intrachloroplastic subdomains leading to the modification of stromal thylakoids, probably significantly affecting the photosynthesis processes that regulate the efficiency of photosystem II. This is partially compensated for by the fact that, at the same time, under normal conditions, the production of peroxide induces the activation of ROS detoxification enzymes. However, a violation of a number of processes, such as the metabolism of accumulation, and utilization and transportation of sugars and starch, is significantly altered, which leads to a shift in metabolic chains. The expected step for further improvement of the applied technology could be both the use of inducible promoters in the expression cassette, and the addition of other genes encoding for hydrogen peroxide-scavenging enzymes in the genetic construct that are downstream in the metabolic chain.

## 1. Introduction

The use of a genetic engineering approach to regulate oxidative metabolism to neutralize and reduce damage during abiotic and biotic stresses was proposed quite a long time ago [1]. Overexpression of various superoxide dismutase genes has proven to be a very interesting and promising strategy. Due to the induction of peroxide, other antioxidant defense enzymes are activated, which leads to an increase in resistance and a decrease in productivity loss under adverse stress conditions such as salt, drought, or cold [1,2,3,4,5], but does not improve plant growth under optimal conditions.

Research in this area can be divided into two fundamentally different, albeit equally substantiated, approaches. The first approach is the concept of saving cells from damage induced by reactive oxygen species due to the formation of direct protection by reducing the amount of ROS in a separate compartment, cell, or tissue. For this, genetic constructs with a wide variety of genes encoding effective enzymes were used in one way or another by cells to neutralize ROS. Researchers have attempted to produce transgenic plants to reduce ROS by heterologous expression of the genes of catalase, ascorbate peroxidase, dehydroascorbate reductase, glutathione reductase, and glutathione-S-transferase, which exhibited altered antioxidant metabolism with effective neutralization of ROS and improved stress tolerance to various unfavorable environmental conditions [1,5,6,7,8,9,10]. Somewhat remote, but close to this concept of indirectly reducing oxidative damage, was the idea of using all kinds of chaperones [11], LEA proteins [12], or osmotically active substances to avoid oxidative damage by reducing the ability of ROS to damage membranes and proteins [13,14]. Another option is the attempt to successfully use the introduction of a number of genes encoding for the ROS neutralization enzymes involving catalase, ascorbate peroxidases, and glutathione reductases [8,15,16]. These enzymes significantly reduce the risk of oxidative damage by converting ROS from oxidizing agents to neutral compounds, in particular, water.

It is known that the generation of superoxide radicals O_2_^−^, HO^−^, and H_2_O_2_ occurs in different cell organelles, which is confirmed by the localization of the corresponding enzymes [17]. The compartmentalization of ROS production in the plant cell determines the biological function of ROS in maintaining balance. Local fluctuations in the concentration of ROS and their accumulation depend on the composition, presence, and activity of antioxidant systems. The main cellular organelles in which ROS are formed are chloroplasts [18,19], mitochondria [20,21], peroxisomes [22], and the apoplast [23]. The production of ROS in chloroplasts is very closely related to the light-dependent reactions of photosynthesis, where the increasing rate of ROS formation serves as a trigger for changes in the impact of abiotic environmental factors, and leads to the regulation and adaptation of metabolic processes [17]. The first enzymatic system that resists the increase in excess active oxygen is a pool of various superoxide dismutases that have organelle-specific localization. Neutralization of ROS is associated with the site of their generation; therefore, chloroplast superoxide dismutases are the determining element of the first stage, leading to the conversion of reactive oxygen species into peroxide, which is then neutralized by other enzyme systems. Enhancement of superoxide dismutase activity through ectopic expression of additional superoxide dismutases, including heterologous ones and those with a different localization within the cell, has been shown repeatedly [24]. Cytoplasmic superoxide dismutase, targeted into the plastid, causes significant changes in the structure of this plastid under normal conditions, but helps neutralize damage during increasing natural stress, restoring both the structure and biochemical balance [25].

Finally, the strategy of neutralization with the formation of H_2_O_2_, the first enzyme in this metabolic chain of neutralization of ROS, and an enzyme such as superoxide dismutase, with the diversity characteristic of eukaryotic cells and different localization [4,26,27], is considered separately. While the product of the functioning of other enzymes is a harmless compound, the product of the functioning of superoxide dismutase is a very toxic component, hydrogen peroxide. However, in addition to the damaging effect on its own intracellular structures and various metabolic processes, hydrogen peroxide also damages various structures of phytopathogens (such as viruses, microorganisms, and fungi), performing a defensive function [28,29]. In addition, this compound plays an important role in stress response signaling [30,31,32,33]. To understand the feasibility of using enzymes involved in different stages of ROS neutralization, it is important to reveal the subtle mechanisms and interactions that are reflected at the level of the structural organization of the photosynthetic machinery, which is responsible for the total productivity of plants, taking into account the peculiarities of the response to the adverse effect of stress.

Therefore, we studied the influence of non-fatal cold damage induced by low positive temperatures and also the change and restoration of the ultra-structural organization of photosynthetic membranes associated with the functioning of PSI and PSII, as well as other chloroplast subdomains after stress exposure. Our special interest was to reveal the changes in fine structural plastid organization, with the aims of addressing the relevance of such a genetic engineering approach and identifying its manifest limitations.

## 2. Results

**Figure 1:**
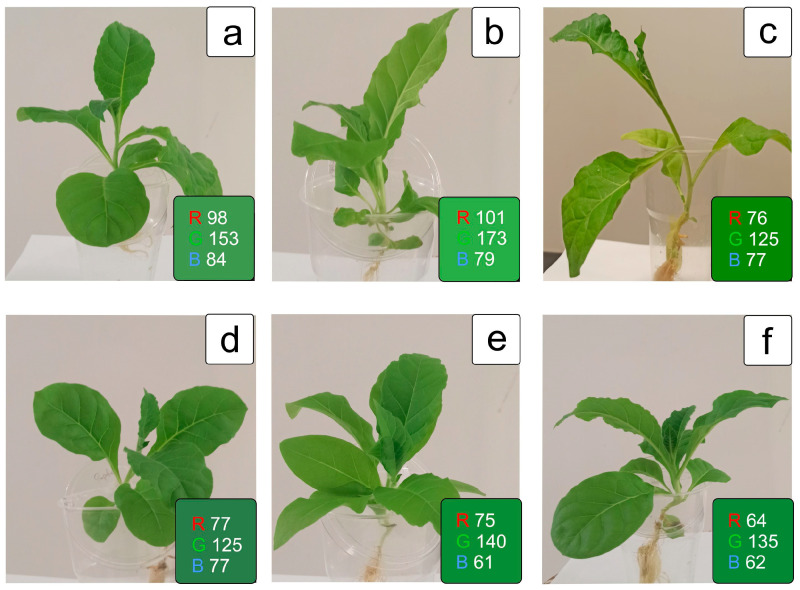
Plants phenotyping and RGB (red, green, blue) reflectance: WT (**a**–**c**) and *FeSOD*-transgenic (**d**–**f**) tobacco plants: normal conditions (NC) at 24 °C (**a**,**d**); at 7 days of cultivation under lowering the temperature to 8 °C (**b**,**e**); as well as a day after the removal of cold stress conditions at 24 °C (**c**,**f**).

The plants of *FeSOD*-transgenic and control initial tobacco lines did not have statistically significant distinctions in the size and habitus of the aboveground part and roots after micropropagation. However, *FeSOD*-transgenic plants had slightly shorter internode lengths and slightly greener foliage under identical culture conditions (Figure 1a,d). A 7-day exposure to a low temperature of 8 °C (Figure 1b,e) and the return to control conditions of 24 °C did not cause visible significant irreversible damage to the leaves or roots of these plants (Figure 1c,f). However, noticeable differences included changes in the shape and color of the leaf blade and slight yellowing in the lower leaves, which is shown in the RGB color indices in each image.

### 2.1. Plant Morphology and Phenotyping

**Figure 2:**
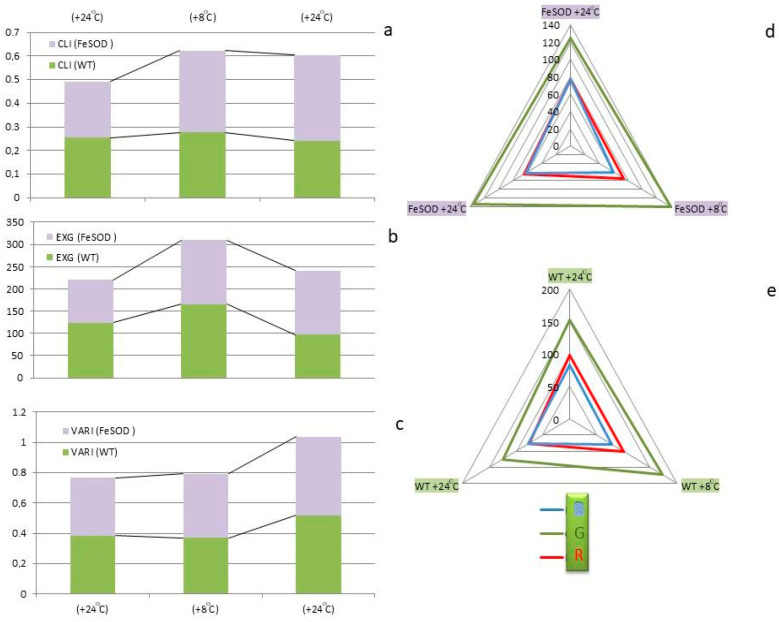
Averaged color phenotyping data and CLI (**a**), EXG (**b**), and VARI (**c**) indices constructed on their basis in leaves of WT and *FeSOD*-transgenic tobacco plants: normal (NC) at 24 °C; after 7 days of cultivation under lowering the temperature to 8 °C; as well as a day after the removal of stress. Numerical color values in red, green and blue (RGB) for WT (**d**) and *FeSOD* (**e**) plants.

A comparative assessment of the state of WT and *FeSOD*-transgenic plants carried out using the calculation of GLI vegetation indices allows us to reliably identify changes in the state of plants after exposure to cold and upon return to optimal conditions (Figure 2). All three indices showed significant differences between the initial state of plants and changes after cold exposure when returning to the optimum. However, VARI was not effective for distinguishing between WT and transgenic plants. Only the EXG index allows one to reliably distinguish the state of transgenic and WT plants under all temperature influences studied.

GLI can only be useful in analyzing differences in plant status under stress conditions and in the initial stages of recovery. If we compare changes in the spectral characteristics of plants under optimal conditions, and after exposure to low positive temperatures and a return to the temperature norm, the most stable indicator was the blue spectrum, which was established for both transgenic and WT plants. However, exposure to low temperature caused a decrease in transgenic plants without affecting WT, while the level of intensively blue was higher in transgenic plants and approached that of WT only at low temperatures and on the first day after. Indicators of intensity in the red spectrum increased with decreasing temperature in both WT and *FeSOD*-transgenic plants, falling lower than in optimal conditions when returning to the optimum after stress. The greatest difference was noted in the color intensity of the green spectrum. Thus, the values of this indicator were more stable in plants expressing *FeSOD*, both under normal conditions and under changing conditions. When exposed to cold at 8 °C and on the first day after exposure, they increased slightly, but did not reach WT values without exposure. Non-transgenic (WT) plants showed a significantly higher sensitivity of color intensity in the green range, which increased markedly at low temperatures and decreased after removal of cold exposure, while the level became comparable to the value in transgenic plants, which were a priori exposed to higher levels of ROS. It can be expected that this method of assessing conditions will sufficiently reflect the response of plants to stress caused by low temperature. Transgenic plants retained their green color and were less sensitive to the effects of cold, which probably correlates with the preservation of the ability to maintain the integrity of the photosystem structures in an intact state.

### 2.2. Antioxidant Enzyme Activity

**Figure 3:**
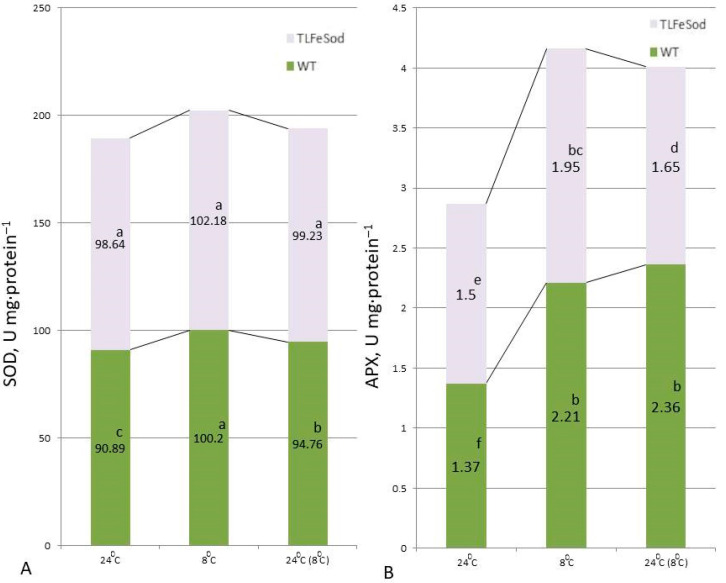
(**A**) Superoxide dismutase and (**B**) ascorbate peroxidase activity in leaves of WT and *FeSOD*-transgenic tobacco plants. Enzymatic activity is presented under normal conditions (NC) at 24 °C; after 7 days of cultivation under of lowering the temperature to 8 °C; as well as a day after the removal of stress. Letters indicate significant differences were determined (*p* ≤ 0.05).

Differences in superoxide dismutase activity were observed in leaves of *FeSOD*-transgenic and WT plants (Figure 3). Regardless of the presence of the applied influence (low temperature) or its absence (control conditions), the activity of superoxide dismutase in *FeSOD*-transgenic plants was two or more higher than that of the control plants. Moreover, this ratio was persisted even when the conditions returned to normal. A trend toward increased activity in response to cold stress was noted in both *FeSOD*-transgenic and WT control plants. It can also be noted that the SOD activity in the leaves of the control and *FeSOD*-transgenic plants after cold exposure decreased to values close to normal, while after cold stress relief, a decrease in SOD activity for transgenes and control plants was observed, but it was weakly pronounced.

Under normal conditions, ascorbate peroxidase activity was higher in *FeSOD*-transgenic plants (Figure 3B). The low-temperature effect was characterized by an almost identical increase in the activity of ascorbate peroxidase in the leaves of both the transgenic and WT lines. On the other hand, when the action of stressors was removed in the WT plants, the ascorbate peroxidase activity remained high, while in *FeSOD*-transgenic plants the activity decreased to nearly the initial values for this line.

### 2.3. Cell Prolifiration and Osmotic Content

**Figure 4:**
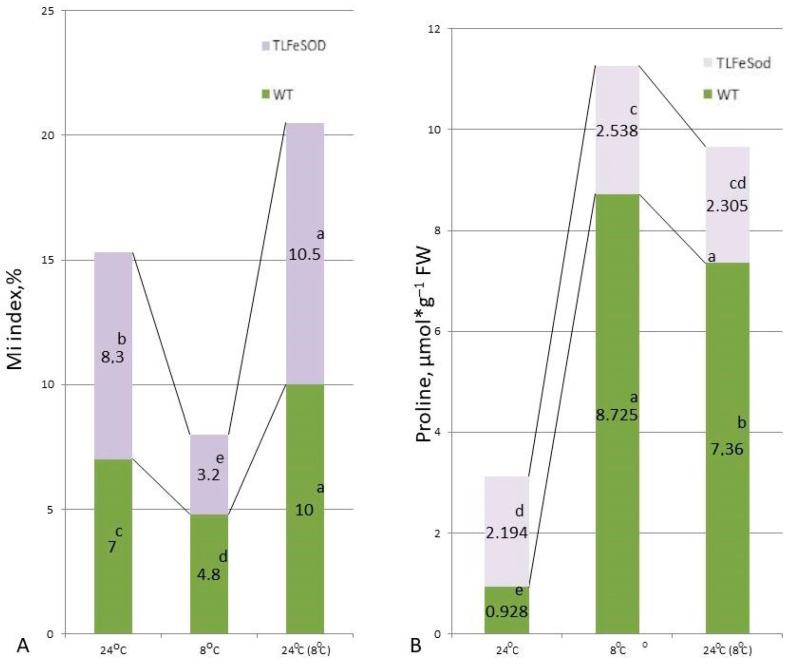
Mitotic index in root apical meristem cells (**A**) and proline content (**B**) in WT and *FeSOD*-transgenic tobacco plants: normal conditions (NC) at 24 °C; after 7 days of lowering the temperature to 8 °C; as well as a day after the removal of stress. Letters indicate significant differences were determined (*p* ≤ 0.05).

The mitotic index of WT tobacco plants was 6.5%. A decrease in the mitotic index by 30% after exposure to low positive temperatures was observed. Return to normal temperature after cold exposure caused a 2-fold increase in the mitotic index (Figure 4A), demonstrating the exit of cells from the compelled G0 state. In *FeSOD*-transgenic plants, the mitotic index under the influence of a low positive temperature of 8 °C decreased by 40%, and the subsequent daily stay of plants at 24 °C after the end of low positive temperatures led to an increase in the mitotic index in relation to the state before cold exposure by 10%, and 3 times more than when exposed to cold (Figure 4A). Thus, a decrease in the number of dividing cells in *FeSOD*-transgenic plants demonstrated their greater susceptibility to cold signaling, a better ability to stop cell division under unfavorable conditions, and an effective recovery of division after the onset of more favorable temperature conditions.

The proline content in the tissues of *FeSOD*-transgenic plants was more than twice the level in WT plants under optimal conditions (Figure 4B). Cold stress caused a noticeable rise in proline content in WT plants (about 8 times) and a noticeably smaller increase in the proline pool in *FeSOD*-overexpressing plants. After cold stress was removed, the decrease in the amount of proline in *FeSOD*-transgenic plants was insignificant and reached the values observed in plants not subjected to stress. At the same time, in WT plants, although they showed a statistically significant decrease in the amount of proline when returning to normal cultivation conditions, the proline content was more than seven times higher than in the absence of cold stress (Figure 4B).

### 2.4. Ultrustructural Analisis of Chloroplast Compartment

**Figure 5:**
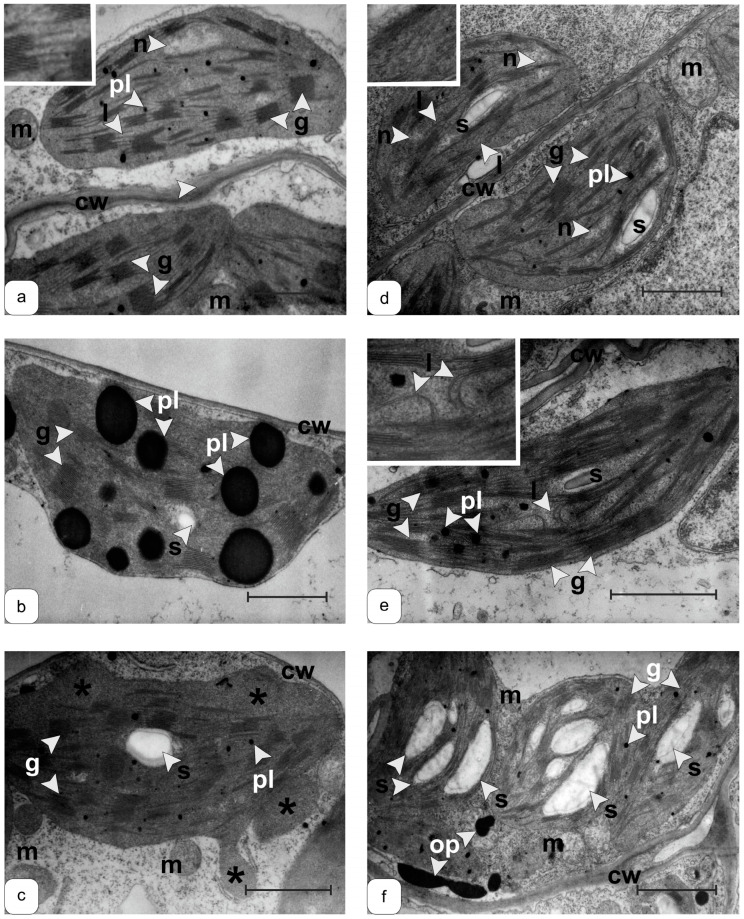
Mesophyll cell chloroplast ultrastructure in tobacco leaves of WT (**a**–**c**) and *FeSOD*-transgenic (**d**–**f**) plants under normal conditions 24 ° C (**a**,**d**), 7 days after low-temperature exposure at 8 °C (**b**,**e**), and one day after recovery following stress removal 24 °C (**c**,**f**). Bar = 1 µm. Legend: pl—plastoglobule; g—grana; s—starch grain; n—plastid nucleoid; m—mitochondria; cw—cell wall, l—lamellae; op—osmiophilic particles; arrow—stromal thylakoids (lamellae); asterisk—residual protrusions of the outer membrane of chloroplasts filled with stroma.

**Figure 6:**
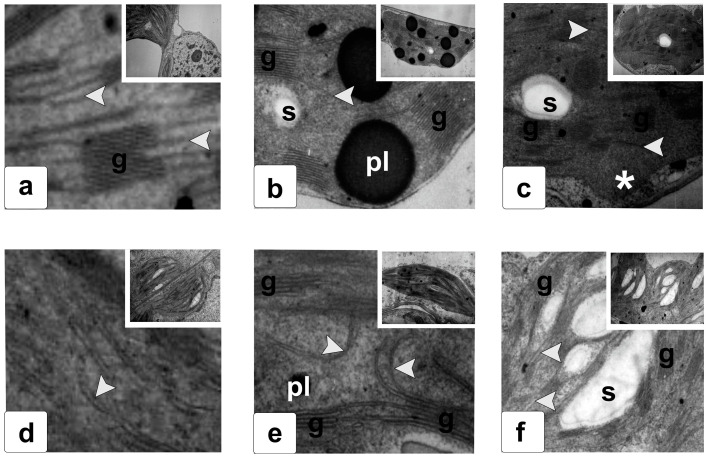
Fragments of plastids from mesophyll cell chloroplast ultrastructure in tobacco leaves of WT (**a**–**c**) and *FeSOD*-transgenic (**d**–**f**) plants under normal conditions (**a**,**d**), 7 days after of low-temperature exposure under +8 °C (**b**,**e**), and one day after recovery following stress removal (**c**,**f**). Legend: pl—plastoglobule; g—grana; s—starch grain; arrow—stromal thylakoids (lamellae); asterisk—residual protrusions of the outer membrane of chloroplasts filled with stroma.

To study ultrastructural damage, characteristic damage to the mesophyll cell plastids of the central part of leaf (Figure 5 and Figure 6) of the WT (Figure 5a–c and Figure 6a–c) and transgenic plants before (Figure 5d–f and Figure 6d–f) and after 7 days of 8 °C conditions (Figure 5b,e low-temperature exposure) (Figure 6b,e), and also one day after the removal of the effects (Figure 5c,f and Figure 6c,f), was revealed.

The ultrastructure in cells of transgenic plants manifested in the mutual arrangement of lamellar subcompartments (Figure 5d and Figure 6d) and an increase in the number and size of plastoglobules (Figure 5d). Stressful effects caused changes in the structure of lamellae, reducing the correctness of their location in WT plastids (Figure 5 and Figure 6).

The cold caused a significant increase in the size of plastoglobules (Figure 6b) in the chloroplasts of WT leaves and their “disappearance” upon stress relief (Figure 6c). In *FeSOD*-transgenic plants’ leaves, an increase in the amount of starch following the removal of cold exposure was observed (Figure 6f). Low temperature made it possible to preserve the structural organization of plastids both under stress and after its removal, demonstrating a higher stability of the functional organization of structures (Figure 5e,f and Figure 6e,f).

### 2.5. Content of Photosynthetic Pigments

**Figure 7:**
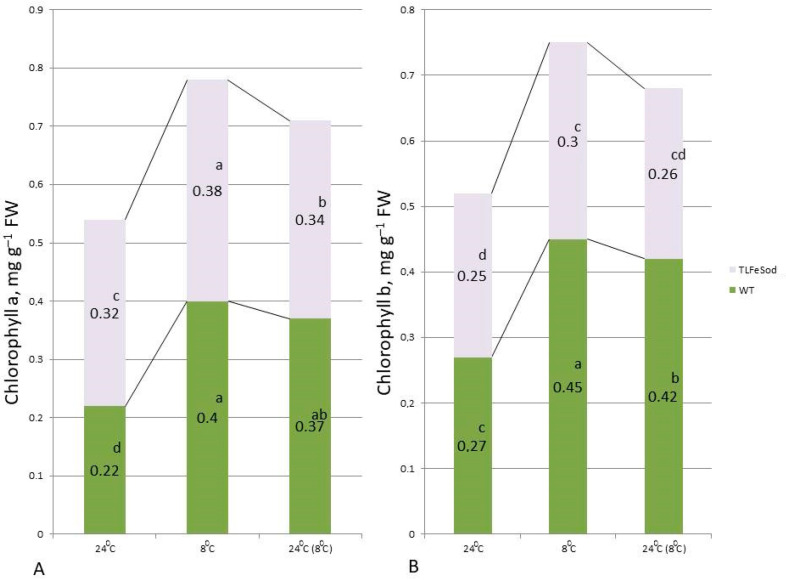
Content of chlorophyll a (**A**) and b (**B**) in leaves of *FeSOD*-transgenic and WT tobacco plants when cultivated under optimal temperature conditions, when the temperature was reduced to a development-inhibiting low positive temperature (8 °C), and a day later when the temperature was restored to optimal values (24 °C). Letters indicate significant differences were determined (*p* ≤ 0.05).

To assess photosynthesis, biochemical indicators such as the content of chlorophylls a and b in leaves, their ratio, and the content of carotenoids are often used. As follows from the data obtained, the content of chlorophylls a and b in the leaves of *FeSOD*-transgenic tobacco plants grown at 24 °C was statistically significantly higher than in WT plants (Figure 7), indicating an intensification of the photosynthetic apparatus formation. After returning to the optimal temperature, the chlorophyll a content in WT plants was higher than in unstressed plants, but lower than at 8 °C (Figure 7). As for chlorophyll b, when exposed to low positive temperatures (8 °C), no significant changes in this indicator were observed in *FeSOD*-expressing plants in comparison with plants not exposed to cold. In the leaves of WT plants, cold caused an increase in chlorophyll b, which decreased slightly after 24 h of stress removal (Figure 7).

After returning to the optimal temperature, the chlorophyll a content in WT plants was higher than in unstressed plants, but lower than at 8 °C (Figure 7). As for chlorophyll b, when exposed to low positive temperatures (8 °C), no significant changes in this indicator were observed in *FeSOD*-expressing plants in comparison with plants not exposed to cold. In the leaves of WT plants, cold caused an increase in chlorophyll b, which decreased slightly after 24 hours of stress removal (Figure 7).

### 2.6. Content of Total Phenolic Compounds

**Figure 8:**
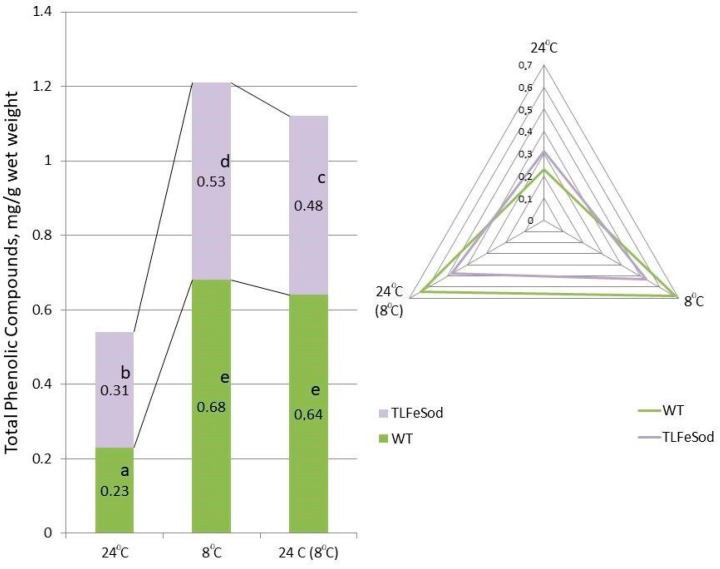
Sum of phenolic compound content in leaves of *FeSOD*-transgenic and WT tobacco plants when cultivated under optimal temperature conditions, when the temperature was reduced to a development-inhibiting low positive temperature (8 °C), and a day later when the temperature was restored to optimal values (24 °C). Letters indicate significant differences were determined (*p* ≤ 0.05).

The sum of phenolic compounds in the leaves of *FeSOD*-transgenic tobacco plants grown at 24 °C was significantly higher than in WT plants, although these differences were not as great as under stress (Figure 8). After exposure to low positive temperatures, the content of phenolic compounds increased: in the leaves of WT plants by almost 3 times, and in the *FeSOD*-transgenic line by 1.8 times. This corresponds to a decrease in sensitivity and an increase in tolerance to this stressor in plants overexpressing Fe-dependent superoxide dismutase.

This corresponded to a decrease in sensitivity and an increase in tolerance to this stressor in tobacco plants overexpressing Fe-dependent superoxide dismutase. When cold stress was removed, insignificant signs of a decrease in the quantity of phenolic compounds were noted in both WT and transgenic plants, which did not reach the values of plants not subjected to stress, while the differences between WT and *FeSOD*-transgenic plants remained.

### 2.7. Carotenoid Content and Lipid Peroxidation Level

**Figure 9:**
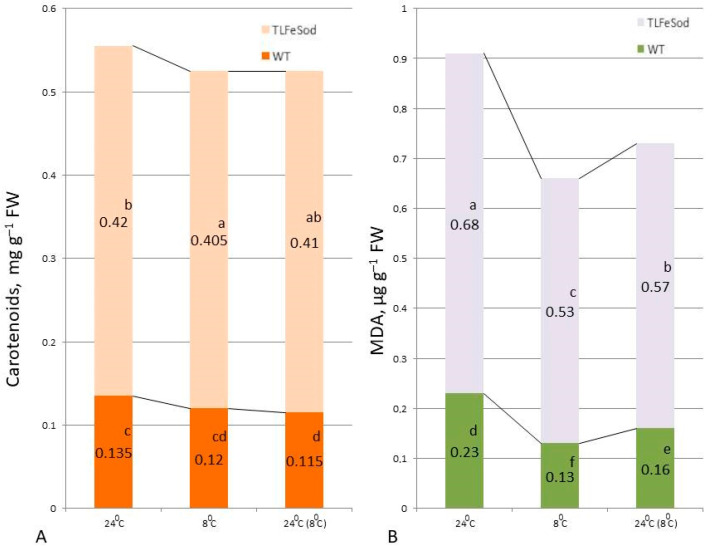
Carotenoid (**A**) and malondialdehyde (**B**) contents in leaves of *FeSOD*-transgenic and WT tobacco plants when cultivated under optimal temperature conditions, when the temperature was reduced to a development-inhibiting low positive temperature (8 °C), and a day later when the temperature was restored to optimal values (24 °C). Letters indicate significant differences were determined (*p* ≤ 0.05).

The carotenoid content in leaves of *FeSOD*-transgenic plants was lower than that of WT plants (Figure 9A). Under the influence of cold, there was a three-fold increase in carotenoid content with subsequent restoration of the level in both *FeSOD*-transgenic and WT plants. After restoration of the temperature regime, a significant trend towards an increase in carotenoids in the leaves of WT plants was observed.

The level of lipid peroxidation (LPO) in plant leaves was assessed by the malondialdehyde (MDA) content. In the leaves of WT tobacco plants grown under normal conditions (24 °C), the MDA content was slightly higher compared to that of the *FeSOD*-transgenic plants (Figure 9B). After exposure to low positive temperatures, it increased in both WT and *FeSOD*-expressing plants, which is more typical for non-transgenic plants. One day after the stress was removed, MDA indicators remained high in WT plants, but decreased significantly in the leaves of *FeSOD*-transgenic plants, indicating a more rapid normalization of this indicator.

### 2.8. The Influence of Low Positive Temperatures on the Cell Cycle

**Figure 10:**
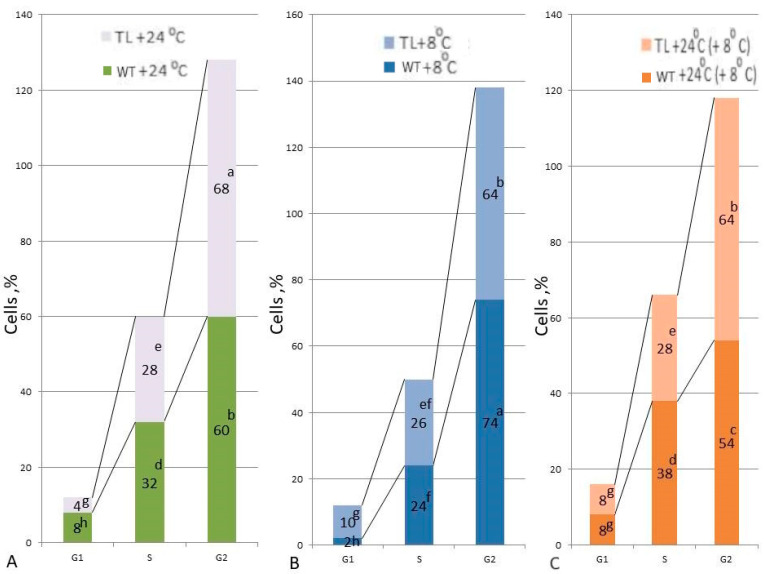
The influence of low positive temperature (8 °C) and restoration of the temperature regime (24 °C) on the distribution of tobacco root meristem cells across phases of the cell cycle. (**A**) temperature (24 °C); (**B**) cold exposure (8 °C); (**C**) restoration of temperature (24 °C); 1—WT (tobacco variety Samsun); 2—*FeSOD*-transgenic tobacco. Letters indicate significant differences were determined (*p* ≤ 0.05).

DNA content was determined by cytophotometry in root meristematic cells of control (WT) and *FeSOD*-transgenic tobacco plants (Figure 10). In both WT and transgenic tobacco plants exposed to low positive temperatures (8 °C), the majority of cells exhibited G2/M phase cell cycle arrest (Figure 10A).

After restoration of the normal temperature regime (24 h, 22 °C), the number of cells in the G2 phase increased in WT plants, while in *FeSOD*-transgenic plants the number of cells in the G2 phase slightly decreased and the number of cells in the S phase increased (Figure 10B). However, in *FeSOD*-transgenic plants, the number of cells in the G2 phase was 15% less than in the WT plants under these conditions. In the WT tobacco plants, the mitotic index (MI) was 6.5%. After exposure to cold stress (8 °C), a decrease in MI by 30% was observed, while restoration of normal temperature conditions (24 °C) after cold exposure increased MI by 2 times (Figure 10C). In the *FeSOD*-transgenic plants, MI after cold stress decreased by 40%, and subsequent daily exposure of plants to 24 °C increased MI by 3 times (Figure 4).

### 2.9. The Determination of ROS in Root Tissue

**Figure 11:**
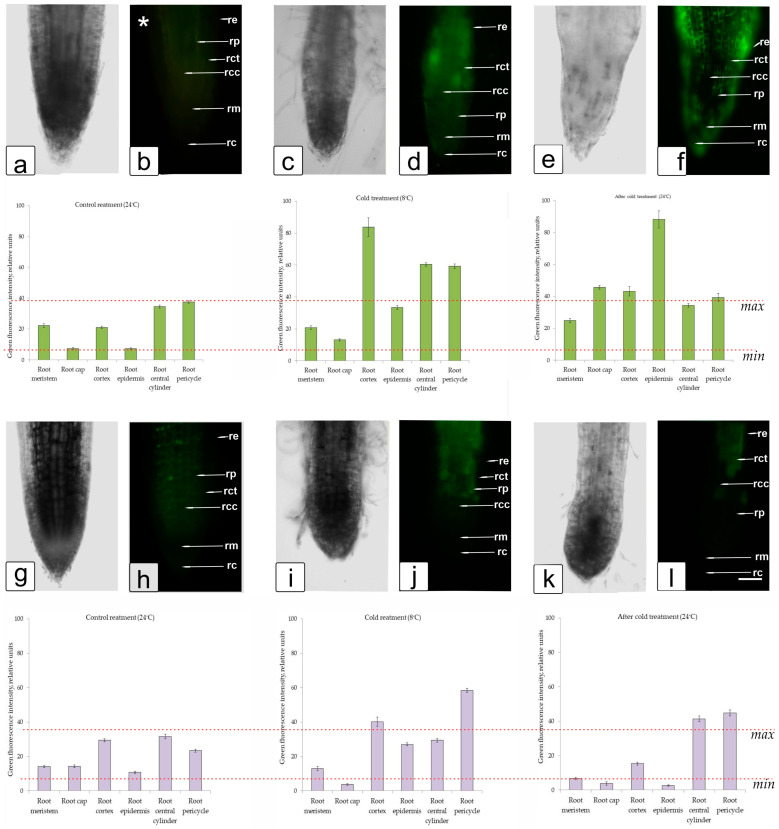
Distributions of ROS+ and ROS in cells in root zones of *FeSOD*-transgenic and WT tobacco plants when cultivated under optimal temperature conditions (24 °C—(**a**,**b**,**g**,**h**)), when reduced to a development-inhibiting low positive temperature (8 °C—(**c**,**d**,**i**,**j**)) and restoring the temperature to optimal values (24 °C—(**e**,**f**,**k**,**l**)). Designations: (**a**,**c**,**e**,**g**,**i**,**k**)—intravital image of the root tip, (**b**,**d**,**f**,**h**,**j**,**l**)—The intensity of Carboxy-H2DFFDA (ROS fluorescence) under the influence of cold stress in WT (**a**–**f**) and *FeSOD*-transgenic tobacco plants (**g**–**l**) indicates a significant difference (*p* ≤ 0.05). Each treatment is accompanied by a histogram showing a quantitative assessment of the intensity of green fluorescence (from RGB image content) in different root tissues: root meristem (rm), root cap (rc), root cortex (rct), root epidermis (re), root central cylinder (rcc), root pericycle (rp). An asterisk marks an image in which the fluorescence intensity has been increased to improve the visibility of root tissue.

Fluorescence due to the marker of reactive oxygen species (ROS) Carboxy-H2DFFDA in root cells of WT tobacco plants when exposed to low positive temperatures (8 °C) was observed more often than under normal conditions (24 °C), and more intense fluorescence predominated (Figure 11).

Single-stained cells were found on the surface of the roots, but the intensity of staining varied between tobacco lines in cells from different zones of the roots. Under cold stress, the most intense ROS staining was observed in the cell division zone in *FeSOD*-transgenic plants, whereas in WT plants, intense fluorescence was observed in the elongation and differentiation zones. After restoration of normal temperature conditions (22 °C), a small number of cells with ROS markers were observed in the roots of *FeSOD*-transgenic plants in the meristematic and elongation zones (Figure 11g–l). In WT plants, more fluorescence of cells stained with a marker for ROS was observed in the cap and meristem zones (Figure 11a–f).

## 3. Discussion

Cold is the most common abiotic stress that suppresses crop growth and productivity. Low positive temperatures affect cellular metabolism and can inhibit the activity of many important enzymes, cell division, and reproduction, and cause membrane disorganization and osmotic imbalance. Ultimately, this can lead to inhibition of growth and even death of plants, especially in such a thermophilic crop as tobacco.

Under abiotic stress, the activity of antioxidant enzymes increases, scavenging reactive oxygen species (ROS), which are potentially dangerous for the normal functioning of living cell systems [34]. Superoxide dismutase is an enzyme that provides the primary stage of ROS scavenging, converting various ROS into hydrogen peroxide molecules that perform regulatory and signaling functions and are subsequently neutralized by other antioxidant enzymes [4,26,34,35]. In photosynthetic organelles, maintaining the ROS balance ensures the metabolic stability of both intraplastid domains and the cells and organism as a whole [4,36,37,38]. In our study, low-temperature stress (8 °C) caused a characteristic reaction: control (WT) plants showed an increase in the total activity of superoxide dismutase. Removing stress after 24 h led to a decrease in activity (Figure 3). This could indicate a positive effect of increasing tolerance due to the functioning of the transgene, at least for the photosynthetic compartment. *FeSOD*-transgenic plants before cold exposure were characterized by an increased level of superoxide dismutase activity (Figure 3A). A significant increase in enzymatic activity was caused by activation of the expression of the entire extensive pool of various resident superoxide dismutase genes due to the stress effect [10,37]. Removal of cold influence resulted in a decrease in superoxide dismutase activity in the *FeSOD*-transgenic plants (Figure 3A), but the levels were significantly higher than the activity in the leaves of control non-transgenic WT plants. It is likely that the decrease in activity may also occur due to changes in the functioning of mitochondrial and cytoplasmic superoxide dismutases [30,37], which remove ROS localized in non-plastid subcellular compartments [36,39].

The activity of ascorbate peroxidase varied significantly [36,39] in *FeSOD*-transgenic and WT plants (Figure 3B). Such differences between them are likely due to increased production of hydrogen peroxide and differences in its compartmentalization. A similar increase in enzymatic activity during cold exposure followed by a decrease in activity can be assessed as very impressive. This indicates that the sensitivity of the pool of superoxide dismutase to external abiotic influences continues to be maintained in *FeSOD*- transgenic plants.

The ultrastructure of photosynthetic plastids is a highly sensitive marker of damage. In the case of structural disturbances, the damages to membrane complexes, which are domains containing lipids, proteins, and individual protein complexes, are visualized [40]. They provide transformations in different parts of the plastid stroma, both near membrane systems and in zones of nucleoids, peripheral membrane formations, and places of deposition and utilization of reserve substances (various types of plastoglobules and starch grains) [37]. In addition, various molecular systems may play important functional roles by ensuring proper compartmentation and regulation of traffic between plastids and other cellular compartments [41,42]. Our study showed that overexpression of Fe-dependent superoxide dismutase led to the expected significant change in chloroplast morphology, and especially to the structural disorganization of stromal thylakoids and disruption of the metabolism of reserve substances (Figure 5). It was previously shown that ectopic overexpression of the *FeSOD* gene is accompanied by tissue-specific changes in the number, size, and morphology of plastoglobules and starch grains in chloroplasts [39]. Apparently, this effect is associated with an induced increase in the content of hydrogen peroxide, which causes a violation of the mobilization and subsequent transport of reserve metabolites to other cells and organs, as well as a limitation of their use [43]. It is also possible that this was due to stress-induced changes in the activity of enzymes involved in these processes [44]. Cold stress causes characteristic transformations in chloroplasts, accompanied by biochemical and structural changes [45,46]. There is also an increase in plastoglobule sizes [36,47], changes in the morphology of the thylakoid system [48], disruption of the plastid shape [49], changes in starch production [43,50], and disturbances in the structural organization and relative position of thylakoid membranes [51], accompanied by an increase in the volume of the intermembrane space [43]. An increase in plastoglobules is accompanied by increased synthesis of carotenoids and a change in the ratio of chlorophylls a and b [52,53]. *FeSOD*-transgenic plants (Figure 5d), which differed from the WT control (Figure 5a), when cultivated under normal conditions (Figure 5d), demonstrated high preservation and even some stabilization of the ultrastructure. Thus, bending of lamellae and invaginations of stromal thylakoids inside the chloroplasts of *FeSOD*-transgenic plants were observed in the absence of stress effects (Figure 5d). Previously, we showed the same changes in the ultrastructure of chloroplasts in transgenic plants [36]. These structures do not disappear under stress, but become less pronounced (Figure 5e). Between the thylakoid complexes of the grana and stroma, a curved but stable parallel arrangement is observed (Figure 5e). It is highly likely that this effect indicates the effectiveness of the transgenesis method used to withstand abiotic stress. The stable structure of thylakoids and, in general, plastids of transgenic plants, is preserved after stress is removed (Figure 5f). At the same time, significant damage remained in the plastids of the WT plants (Figure 5c). Changes in the shape and relative position of stromal thylakoids, which, according to our data, are most sensitive to the manifestation of FeSOD functioning, are often observed when photosystem II is damaged [39]. When exposed to cold (8 °C), the plastid compartment in WT plant cells underwent significant modification (Figure 6a,b). A change in the shape of plastids was observed, specifically, a sharp hypertrophic increase in the size of plastoglobules. There was also a decrease in the number of stromal thylakoids and the appearance of a curved shape, which is not typical for normal thylakoids. One day after removal of the effect, the shape of the plastids of the control plants did not return to normal, retaining significant protrusions, and retaining invaginations filled with stroma and ribosomes in the places of plastoglobule disintegration (Figure 6b). An increase in the number and size of plastoglobules is a common response to exposure to cold [54] and other stresses [55]. In our earlier work with *FeSOD*-transgenic tobacco plants, we showed that in cases where superoxide dismutase activity is not supported by activation of ascorbate peroxidase, the size of plastoglobules increases significantly [39]. Thus, it can be assumed that the increase in plastoglobules is directly related to the increase in the ROS pool. Similar responses to drought or severe light stress have been observed in other plants [56]. The study of plastoglobules has recently attracted much attention, primarily due to the accumulation of carotenoids, various quinones, and phytol and tocopherol esters in these lipid conglomerates [57], as well as the special role of plastoglobules in redox and photosynthetic regulation [57]. The system of organization of the internal space of chloroplasts in *FeSOD*-transgenic plants was changed (Figure 6d), which most clearly affects the somewhat chaotic ultrastructure of stromal thylakoids (lamellae). The cold influence did not cause significant damage (Figure 6e). There was no increase in the size of plastoglobules or their number. One day after the cold exposure was removed; the plastids had a normal lens-shaped shape (Figure 6e). The ultrastructure of grana and stroma thylakoids also corresponded to those in WT plants (Figure 6a), indicating a prolonged effect of FeSOD expression. An increase in starch content can also be noted (Figure 6e), which may indicate either an increase in the intensity of functioning of photosynthetic systems and the accumulation of reserve metabolites, or indicate a slowdown in the mobilization and transport of sequestered reserves.

One of the main and important metabolic processes in plants is photosynthesis, which provides them with substrates and energy [58]. In our study, a decrease in temperature when growing WT and transgenic tobacco plants in vitro did not affect the formation of chlorophylls in them, this indicated the absence of a stress response in these plants. A similar result was observed in *Arabidopsis thaliana* Heynh. (L.) after exposure to low positive temperatures, where no significant changes in the content of photosynthetic pigments were also detected [59].

Carotenoids are important components of the plant pigment system. Their functional role is associated with the processes of absorption of light energy, as well as participation in the protection of cells from stress [60,61]. An increase in carotenoid content is observed when tobacco plants are exposed to low temperatures [59]. In our work, the same stress response occurred in WT and *FeSOD*-transgenic tobacco plants, based on the accumulation of carotenoids in them as one of the components of cell protection from stress [61]. However, when the stress treatment was removed, *FeSOD*-transgenic plants quickly returned to a higher carotenoid content compared to WT plants (Figure 9A).

The lipid peroxidation (LPO) level, assessed by the amount of malondialdehyde (MDA), is an indicator of the physiological state of plants, and its changes are considered to be a criterion for the presence or absence of a stress response in them to the influence of various exogenous factors [62]. After exposure to low positive temperatures (8 °C), MDA increased in both control plants (WT) and the *FeSOD*-transgenic line. Unlike WT, the transgenic line also showed a significant decrease in the amount of MDA after transfer to control temperature conditions (Figure 9B). The quantitative increase in most antioxidant enzymes, as well as proline and phenolic compounds, in both transgenic and control plants, is a response to stress. However, a significant decrease in MDA in the transgenic line after transfer to normal conditions indicates their antioxidant role. Consequently, constitutive expression of the *FeSOD* gene in tobacco plants contributed to an increase in their resistance to low positive temperatures.

When adapting to stress, plants synthesize low-molecular-weight plant antioxidants, including various phenolics. The total content of phenolic compounds in plant tissues can serve as a criterion for potential resistance to stress [63]. In our experiment, after exposure to low positive temperatures, the total phenolic compounds increased in both *FeSOD*-transgenic and WT plants, which indicated the activation of the biosynthesis of these low molecular-weight antioxidants, which is more pronounced in WT tobacco plants. An increase in the accumulation of polyphenols under conditions of plant adaptation to low temperatures was also observed in other plants [63].

In plants, exposure to stress is often accompanied by a decrease in growth and arrest of the cell cycle M → G1 → S → G2, which is associated with the cell exiting the cycle immediately after division with the transition from telophase to the resting state → G0. Cell cycle disturbances are characteristic of many stress influences and are a reaction of plant cells to temperature stress, dehydration, and salinity [64,65,66]. A study of the cell cycle of the tobacco root meristem showed that exposure to low positive temperatures and subsequent restoration of the temperature regime have different effects on the distribution of cells across periods of the cell cycle (Figure 10A,B). Cytophotometric determination of the dynamics of cell progression through the cycle showed that under the influence of low positive temperatures in the root meristem cells of both WT and *FeSOD*-transgenic tobacco plants, G2/M was blocked (Figure 10A). Apparently, cold, being a cytostatic factor, partially blocked the entry of cells into mitosis. A possible reason for this could be the destruction of microtubules, which led to cytoskeletal disorders in interphase cells [66]. The number of G2 cells during blocking in *FeSOD*-transgenic plants decreased, while the number of cells in the G2 phase during cold exposure in WT plants increased (Figure 10A), which is associated with a change in the duration of the cell cycle phases.

Changes in mitotic activity are expressed by the mitotic index (MI) and are the main characteristic of the proliferative activity of plant cells. A decrease in MI after cold exposure indicates a block in the entry of cells into mitosis, and in transgenic tobacco this process is more pronounced. Meristematic cells in the root apex of *FeSOD*-transgenic tobacco plants were highly sensitive to cold stress: cold exposure blocked proliferative activity. This reaction can be considered as an adaptive response to stress, which minimizes the possible consequences of cytoskeletal disorders in dividing cells and, thereby, prevents the imbalance of the genomes of transgenic plants in mitosis [67]. Returning *FeSOD*-transgenic tobacco plants to normal temperature normalized the proliferative activity of root meristem cells (Figure 10C). A higher level of the mitotic index upon restoration of the temperature regime (24 °C) after cold exposure (8 °C) indicated a greater tolerance of *FeSOD*-transgenic plants to stress compared to WT plants (Figure 10C). This is probably ensured by finer regulation of the sensitivity of division arrest and the subsequent compensatory exit of cells from the G0 phase after the arrest is lifted [68]. The mechanisms of sensitivity, correct signaling, and the formation of responses to cold are shown by the activation of DREB/CBF and other groups of transcription factors, such as MYB, NAC, and ZFP. Cold also induces the expression of genes encoding signaling proteins such as RLK, RLCK, CDPK, and MAPKK [69]. It is likely that an increase in ROS in transgenic plants, without inducing a calcium signal or simultaneously with it, significantly affects the stages of the cell cycle under cold influences, inducing effective arrest of the cell cycle and exit from it through the triggering of protective machinery [70,71]. Thus, *FeSOD*-transgenic plants acquire a new useful property, a kind of “grafting” or cellular memory, using evolutionary mechanisms that guaranteed survival in the long evolutionary process of the formation of modern plants [72].

ROS indirectly controls the cell cycle, cell division, cell expansion, and cell death. An increase in ROS content in plants in response to abiotic stressors has been noted by many researchers. At the same time, ROS are considered simultaneously as markers of a stress state and as signaling mediators necessary for the development of an adaptive response [73,74]. ROS production and ROS-related signal transduction influence stress-induced bioenergetic processes by modifying the structural organization of mitochondria [75,76,77]. This causes a change in metabolism through the regulation of signaling in all aspects of plant growth and development in various organs and tissues [78,79]. ROS have a modulating effect on such processes of ontogenesis and cell growth as the cell cycle, cell division, reproduction, and cell death, through an indirect effect on the transport of phytohormones between plant tissues and organs [34,80]. ROS also influence redox signaling between organelles and the nucleus [81,82,83].

The accumulation of the fluorescent ROS marker Carboxy-H2DFFDA in root cells under the influence of low temperatures (8 °C) indicated a disruption of ROS homeostasis in individual cells and root tissues in both *FeSOD*-transgenic and WT plants, which could lead to the local triggering of programmed cell death (PCD) [84], and when adaptive mechanisms are triggered, cause the formation of new roots that are more resistant to unfavorable conditions [85]. In WT plants, exposure to low temperatures showed an expansion and high intensity of localization on root sections of the fluorescent ROS marker Carboxy-H2DFFDA, which covered wider areas, indicating a wide area of activation of oxidative damage and an increase in ROS content in these cells (Figure 11d,f). In the roots of WT-tobacco plants growing under optimal conditions (24 °C), the Carboxy-H2DFFDA marker was almost not detected (Figure 11b). In *FeSOD*-transgenic plants under optimal conditions (24 °C), a slight increase in the level of ROS and small local zones with increased fluorescence, presumably in the epidermis zone, were identified (Figure 11g,h). At 8 °C, and one day after returning to optimal conditions (24 °C), in *FeSOD*-transgenic plants the most intense ROS staining was in the cells of the cortex and the central cylinder of the differentiation zone (Figure 11i–k). The fluorescence intensity in the root cells of *FeSOD*-transgenic plants was significantly lower than in WT plants, which indicated greater tolerance to cold, at least to the effects of low positive temperatures (8 °C). These data correlated with an increase in cold adaptability in transgenic alfalfa and white clover plants [6,86].

Constitutive activation of adaptive antioxidant mechanisms was carried out in earlier works [1,6], which appears to be a useful indirect systems approach to increasing acclimation to cold stress, as well as other stresses associated with oxidative damage [87]. This process is ensured by the induction of a permanently increased content of ROS in cellular organelles and the cell as a whole, which can lead to a completely insignificant deterioration in plant performance under optimal conditions, but bring obvious benefits when stress occurs.

## 4. Materials and Methods

### 4.1. Plant Material and Growth Conditions

Transgenic and non-transgenic plants of *Nicotiana tabacum* L. cv Samsun were used in the study. Non-transgenic wild-type (WT) plants were used as controls. Transgenic plants were obtained earlier [36,88]. They were transformed with the Fe-containing superoxide dismutase (*FeSOD*) gene from *Arabidopsis thaliana* L. under the control of a standard CaMV 35S promoter [88]. The synthesized enzyme was targeted into the plastid due to a fused signal sequence from the pea ribulose bisphosphate carboxylase/oxygenase gene [1].

Propagated *FeSOD*-transgenic and control WT tobacco plants were planted in containers filled with perlite and ½ MC liquid nutrient medium and adapted to liquid culture for 2 weeks. Plants were grown under controlled conditions at illumination of 100 µmol m^−2^ s^−1^, temperature 24 °C, and air humidity 60–70% in a KBW-240 growth chamber (Binder Gmbh, Tuttlingen, Germany). For the experiments, uniform plants were selected in the vegetative growth phase at the age of 14 days, having a developed root system and 5 fully formed leaves. A total of 18 *FeSOD*-transgenic and control, non-transgenic tobacco plants were selected. Then, 12 plants of each variant were subjected to cold stress. Cold stress was simulated by exposure at 8 °C for 7 days in a growth chamber. This regime of cold exposure (8 °C) was used due to the need to preserve the functional activity of the photosynthetic apparatus of the studied plants and the fact that such a temperature does not lead to plant death in tobacco. Next, 6 plants of each variant were returned to optimal conditions (24 °C) after exposure to cold. Thus, the following were compared: (1) plants grown in optimal conditions (24 °C); (2) plants subjected to cold stress (8 °C); and (3) plants returned to optimal conditions (24 °C). Three randomly selected plants from each treatment were used for analysis. After completion of the experiment, the plants were photographed and the images were scanned. For biochemical studies, tissue samples were collected, fixed with liquid nitrogen, and stored at −70 °C. For electron and light microscopy, standard methods for intravital ROS detection were used.

### 4.2. Phenotyping of Tobacco Plants for Determination of Calculated Quantitative Indices Based on Leaf Color

Intravital determination of the spectral characteristics of tobacco plants was carried out immediately after removal from the growth chamber after cultivation in different temperature conditions. Three randomly selected plants of each variant were used for evaluation. The indices were calculated based on the average values obtained when scanning plants based on RGB image analysis. To obtain images, the Phenoscanner Synergotron ISR02-01 (ISR, Moscow, Russia) was used. To assess the condition of plants, indices were calculated based on those proposed earlier. The following indices were used for the calculation: VARI—algorithms for remote estimation of the proportion of vegetation [89]; EXG—color indices for lighting conditions for plant identification [90]; and GLI—documentation of grazing impacts on wheat [91].

### 4.3. Antioxidant Enzyme Activity

Estimation of the activity of antioxidant enzymes was carried out in tissue samples from the leaves of control and transgenic plants under various experimental conditions, as mentioned above. To determine the activity of superoxide dismutase (SOD), 0.5 g excised leaves were ground in a mortar with 4.5 mL of chilled 30 mM K/Na phosphate buffer (pH 7.4) containing 0.1 mM EDTA and 2% polyvinylpyrrolidone. The homogenate was filtered through nylon tissue and centrifuged at 11,000× *g* for 20 min. The supernatant, diluted 20 times, was used as an enzymatic extract to determine the activity of SOD. Superoxide dismutase activity was determined by photoreduction of p-nitro-blue tetrazolium chloride (NBT) in the presence of riboflavin and methionine according to [92] with some modification. The reaction mixture (3 mL) contained 1.3 μM riboflavin, 13 mM methionine, and 63 μM NBT in 0.05 M K/Na phosphate buffer with 0.10 mM EDTA, pH 7.4, and 0.1 mL of enzymatic extract. Samples were illuminated for 6 min. The measurements were carried out on a Hitachi 557 spectrophotometer (Hitachi, Tokyo, Japan). One unit of SOD activity was defined as the volume of the enzyme extract that caused 50% inhibition of NBT photoreduction at 560 nm.

The activity of ascorbate peroxidase was assayed as the decrease in absorbance at 290 nm due to ascorbic acid oxidation [93]. The reaction mixture for detection APX activity contained 25 mM K/Na phosphate buffer (pH 7.4), 0.5 mM ascorbic acid, 0.1 EDTA, and enzymatic extract. The reaction was initiated by adding 0.1 mL of 0.1 mM hydrogen peroxide. The obtained results of all enzymatic assays were calculated as the rate of enzyme activity per gram of leaf fresh weight. The values reported in a radar chart (Excel 2010) (Microsoft, Washington, DC, USA) are the means from 5 replicates. Data analysis was performed using one-way analysis of variance (ANOVA).

### 4.4. Transmission Electron Microscopy

The central fragments of the leaf blade were fixed for electron microscopic analysis. Cut pieces of 1 mm^3^ were immersed in cooled 0.1 M phosphate buffer, then fixed in a 2.5% solution of glutaraldehyde in 0.1 M phosphate buffer, pH 7.4, with the addition of sucrose 0.15 mg mL^−1^ in for 4–8 h in the refrigerator at 4 °C. After this procedure, the samples were washed with 0.1 M Sorensen buffer and fixed in a 1% solution of osmium tetroxide (OsO4) for 2 h at 4 °C. The material was dehydrated in ethanol solutions of increasing concentration at 4 °C. The accessions were then transferred to propylene oxide. A mixture of epon, araldite, and propylene oxide was used in the following concentrations: 1/5; 2/3; 1/1; 3/2; 5/1 (40’ each). The material was transferred into flat plastic molds containing the resin with a catalyst. The polymerization was carried out for 24 h at 45 °C, and then for 24 h at 56 °C. The ultrathin slices were mounted on copper grids with a formvar substrate. Next, the sections were contrasted with 1% uranylacetate (30 min), then washed and contrasted with a solution of lead citrate (15 min) and washed thoroughly in distilled water. After drying, the preparations were analyzed in a Hitachi H-500 electron transmission microscope (Hitachi, Tokyo, Japan) at a working magnification of ×5000–10,000. The resulting images were scanned using EpsonPerfection 3170 (Seiko Epson Corporation, Shinjuku, Tokyo, Japan) at a resolution of 600 dpi. For image processing, MicrosoftPhotoEditor (Microsoft Corporation, Redmond, WA, USA) and CorelDRAW programs (CorelDraw, Ottawa, ON, Canada) were used.

### 4.5. Photosynthetic Pigments Determination

Plant leaf fragments were homogenized in 96% ethanol and the samples were centrifuged for 5 min at 13,260× *g*. The chlorophylls a and b and carotenoid content were determined in the supernatant liquid using a spectrophotometric method on a Genesys 10 UV device (Termo Electron Corp., Waltham, MA, USA) and calculated as mg g^−1^ fresh weight [94].

### 4.6. Total Phenolic Compound Determination

Phenolic compounds were extracted from fresh plant material with 96% ethanol at 45 °C for 45 min [95]. The homogenate was centrifuged for 2 min, 13,260× *g*; the supernatant was separated and used to determine the content of the total soluble phenolic compounds by the spectrophotometric method (Genesys 10 UV, Termo Electron Corp., Waltham, MA, USA) using a Folin–Ciocalteu reagent at 725 nm [96]. Gallic acid was used to construct a calibration curve. The content of phenolic compounds was expressed in mEq. gallic acid g^−1^ fresh weight (mg GAE g^−1^ fresh weight).

### 4.7. Lipid Peroxidation Determination

To determine lipid peroxidation, the content of malondialdehyde (MDA) was measured using a reaction with thiobarbituric acid (TBA) [97]. Plant material was homogenized in 0.1 M Tris-HCl buffer (pH 7.5) containing 0.35 M NaCl. A 0.5% solution of TBA in a 20% aqueous solution of trichloroacetic acid was added to the homogenate, incubated at 95 °C for 30 min, and cooled, and the optical density of the solution was measured at 532 nm. To calculate the MDA content (μmol g^−1^ fresh weight), the molar extinction coefficient (1.56 × 10^−5^ cm^−1^ M^−1^) was used [98].

### 4.8. Cytophotometric Analysis of DNA Content in the Nuclei

For cytophotometric analysis, root tips (3–4 mm) of control and transgenic plants were fixed in a mixture of ethanol and acetic acid (3:1 ratio) for 3 h. The root meristem zone (up to 1–2 mm) was separated and placed for 2 h at 37 °C in a macerating mixture containing 0.4% cellulase (Sigma-Aldrich Co., St. Louis, MO, USA) and 0.4% pectinase (Merck, Darmstadt, Germany), then stained using the Feulgen method (Schiff reagent, Merck) for 2 h (hydrolysis time in 5N HC1 at 22 °C, 40 min) and permanent preparations were prepared. To determine the DNA content, the nuclei of meristematic cells were measured using an SMP-20 Opton cytophotometer (Carl Zeiss, Obercochen, Germany) with a 16 × objective, 10 × eyepiece, and 0.16 mm probe. The nuclei of meristematic root cells at the telophase (2C) or metaphase (4C) stages were used as a standard. The sample for each experimental point was at least 300 nuclei from cells of 10–12 roots. The data were analyzed in the Statistics 6.0 program.

### 4.9. Fluorescence Microscopy for ROS Detection in Root Apex

The root tips (5 mm) of tobacco plants were separated in a drop of fresh solution (1/2 Murashige and Skoog) with a sharp razor immediately after removal from the growth chamber and placed on glass slides in a drop of water, 5–10 pieces per slide, at a rate of 2–3 roots per 1 drop. For intravital visualization of ROS in cells, an aqueous solution of Carboxy-H2DFFDA (Thermo Fisher Scientific Inc., Waltham, NY, USA) was used at a concentration of 25–50 nM; the incubation time was 30 min, followed by washing 3 times in distilled water. Then the living roots were placed in a drop of the incubation mixture on a glass slide. Intravital preparations of root tips were analyzed using an Olympus BX51 microscope (Olympus Corp., Tokyo, Japan), objective ×10, at a wavelength of 490 nm. Typical images were obtained using a Color View II digital camera (Soft Imaging Systhem GmbH, Munster, Germany). RGB images were used to evaluate and calculate the glow intensity in the green range. Histograms show means and significant errors.

### 4.10. Statistical Analysis

All variants of the described experiments and analyses were carried out in triplicate. Statistical processing was carried out using SigmaPlot 12.3 (Systat Software Inc., Chicago, IL, USA) and Microsoft Excel (Microsoft, Washington, DC, USA).

To visualize the data, we used a normalized stacked histogram, which is used to highlight the row total for each category. Statistically, for this type of histogram we do not imply an indication of the error of the mean; the differences are indicated by letters corresponding to the differences established for classical calculations using the Duncan criterion. Experimental data were assessed at 5% significance level using the analysis of variance (ANOVA) and Duncan’s multiple range tests with AGROS software (version 2.11, RSAU-MTAA, Moscow, Russia). In the figures, identical letters indicate indicators that do not differ; if there are significant differences, the letters differ. In the case of the presence of two or more letters, no differences were observed, with the indicators indicated by the corresponding letters. Means followed by the same letter are not significantly different at α = 0.05 according to Duncan’s multiple range test.

## 5. Conclusions

Oxidative stress caused by reactive oxygen species is a general biological highly sensitive system for perceiving and overcoming a large number of negative abiotic factors limiting the efficient productivity of crops. ROS-dependent induction of oxidative damage can be used as an effective trigger to initiate non-specific protection in plant cells and tissues. Plants are often potentially able to withstand various specific (toxic, osmotic) factors of abiotic stress, but do not have (or lose) sufficient fine tuning or specific sensitivity to form an adequate effective response. In this work, we confirm that one of the possible approaches is the formation of effective protection of photosynthetic structures due to the insertion of the foreign *Arabidopsis thaliana FeSOD* gene into the tobacco genome, under the control of the constitutive promoter and equipped with a signal sequence targeting the protein to plastid, which can effectively increase ROS, enhancing adaptability. The increased enzymatic activity of superoxide dismutase in the plant plastid of transgenic tobacco cells enables the plant to tolerate the oxidative factor of environmental stresses scavenging ROS due to the activation of protective programs. Increasing the pool of hydrogen peroxide prevents significant damage to the cell ultrastructure, and also promotes easier recovery after stress relief. Obviously, this cannot affect metabolism and, when grown under normal conditions, somewhat disrupts the arrangement of intrachloroplast subdomains. This leads to modification of stromal thylakoids, likely significantly modifying the photosynthetic processes that regulate the efficiency of photosystem II. This is partially compensated for by the fact that, at the same time under normal conditions, the production of hydrogen peroxide induces the activation of ROS detoxification enzymes. Presumably, a number of processes are significantly disrupted, such as the metabolism of accumulation, and utilization and transport of sugars and starch, which results in a shift in metabolic chains. Expected steps for further improvement of the technology used are the use of inducible promoters in the expression cassette and the addition to the genetic construct of other genes encoding enzymes that capture hydrogen peroxide, which are located downstream in the metabolic chain, as has been conducted in a number of studies.

## Data Availability

Data are contained within the article.
